# Tear Proteome Revealed Association of S100A Family Proteins and Mesothelin with Thrombosis in Elderly Patients with Retinal Vein Occlusion

**DOI:** 10.3390/ijms232314653

**Published:** 2022-11-24

**Authors:** Alexander Stepanov, Svetlana A. Usharova, Kristina A. Malsagova, Larisa K. Moshetova, Ksenia I. Turkina, Arthur T. Kopylov, Anna L. Kaysheva

**Affiliations:** 1 Biobanking Group, Institute of Biomedical Chemistry, 10 Pogodinskaya Str., Bld. 8, 119121 Moscow, Russia; 2 Russian Medical Academy of Continuous Professional Education, Ophthalmology Department, 2/1 Barrikadnaya Str., Bld. 1, 125993 Moscow, Russia

**Keywords:** retinal vein occlusion, retinal hemostasis, S100A protein, mesothelin, inflammation

## Abstract

Tear samples collected from patients with central retinal vein occlusion (CRVO; n = 28) and healthy volunteers (n = 29) were analyzed using a proteomic label-free absolute quantitative approach. A large proportion (458 proteins with a frequency > 0.6) of tear proteomes was found to be shared between the study groups. Comparative proteomic analysis revealed 29 proteins (*p* < 0.05) significantly differed between CRVO patients and the control group. Among them, S100A6 (log (2) FC = 1.11, *p* < 0.001), S100A8 (log (2) FC = 2.45, *p* < 0.001), S100A9 (log2 (FC) = 2.08, *p* < 0.001), and mesothelin ((log2 (FC) = 0.82, *p* < 0.001) were the most abundantly represented upregulated proteins, and β2-microglobulin was the most downregulated protein (log2 (FC) = −2.13, *p* < 0.001). The selected up- and downregulated proteins were gathered to customize a map of CRVO-related critical protein interactions with quantitative properties. The customized map (FDR < 0.01) revealed inflammation, impairment of retinal hemostasis, and immune response as the main set of processes associated with CRVO ischemic condition. The semantic analysis displayed the prevalence of core biological processes covering dysregulation of mitochondrial organization and utilization of improperly or topologically incorrect folded proteins as a consequence of oxidative stress, and escalating of the ischemic condition caused by the local retinal hemostasis dysregulation. The most significantly different proteins (S100A6, S100A8, S100A9, MSLN, and β2-microglobulin) were applied for the ROC analysis, and their AUC varied from 0.772 to 0.952, suggesting probable association with the CRVO.

## 1. Introduction

Retinal vein occlusion (RVO) is an ocular disease with an incidence rate of 4.6% in patients older than 80 years and 0.77% among patients younger than 60 years [1,2]. The disease is fraught with a risk of blindness or severe decrease of visual acuity since the entire retina is affected, especially in older patients [2,3]. As RVO progresses, the retina suffers ischemic conditions upstream of the occlusion localization, which entails escalation of the inflammatory reaction [4].

The prevalence of chronic eye diseases is associated with aging [5,6]. The tacit assumption is that tear proteome is characterized by the age-associated gradual increase of inflammatory proteins and immune response cytokines. Several cytokines, including interleukin-6 (IL-6) and VEGF (vascular endothelial growth factor), are known to be associated with RVO, but still contribute little in the understanding of RVO pathology. The high interindividual variability of cytokines and their sensitivity to epigenetic background are by far the most challenging problems limiting clinical and proteomic studies [7,8]. A comparative tear proteomic study of 30 RVO patients with age-related macular degeneration demonstrated proteome enrichment by complement factors (C3 and C9), clusterin (CLU), S100A9, and S100A8 proteins [9]. A deficiency of glucose-6-phosphate dehydrogenase (G6PD) serum level has been correlated with a risk of CRVO (central retinal vein occlusion) due to the increased vulnerability to oxidative stress [10]. The S100A12 was also considered as a contributor to the inflammation response of the ischemic condition. In an animal model of CRVO, a five-fold increase of S100A12 was found in retinal ganglion cells and their dendritic processes [11]. The abundance of S100A family proteins suggests actively ongoing inflammation, followed by the RVO and the consequent ischemic condition [12]. However, neither of these studies yielded a rational molecular basis of CRVO.

Another side of eye-related proteomic investigation is focused on mitochondria-associated proteins. Proteomic investigation after laser-induced retinal artery occlusion (RAO) relevant to clinical manifestation indicated an increased Bax protein (also known as Bcl-2-associated X protein, or bcl-2-like protein 4), and simultaneous elevation of cytosolic cytochrome *c* and caspase-9, which suggests severe oxidative stress accompanied by the mitochondrial dysfunction and pro-apoptotic events [13]. Dysfunction of mitochondrial ATP generation was also found in the proteome of retinal pigment epithelium obtained from patients with age-related macular degeneration, and in in vitro models of human embryonic stem cells [14,15,16]. Notwithstanding this, dysregulation of mitochondria functioning reflects the consequence of CRVO but does not determine the root of pathology.

Proteomic study of tear fluid is a challenging task due to the low amount of available biomaterial. Despite the fact that modern approaches can achieve a proteome size of more than 3000 proteins for a single run [17,18], the obtained information is not highly promising and carries no information regarding the discovery of clinically relevant biomarkers. One of the critical issues is the standardization of sample collecting and requirements of storage, which affects total protein concentration in the sample [19]; another challenge is the high sensitivity of proteomics to individual variability. The latter causes poor identification of well-known RVO-associated markers such as soluble ICAM1, which is generally associated with capillary blood velocity in patients with branch and central retinal vein occlusion [20,21], whereas other proteins of particular interest are mostly characterized by inflammatory and immune-response activities [22].

This study aimed to determine whether is it possible to distinguish patients with CRVO by their tear proteome. To gain a better understanding of molecular pathways associated with CRVO, we conducted a quantitative proteomic measurement and complemented it with mapping of disease-related protein–protein interactions.

## 2. Results

**Figure 1:**
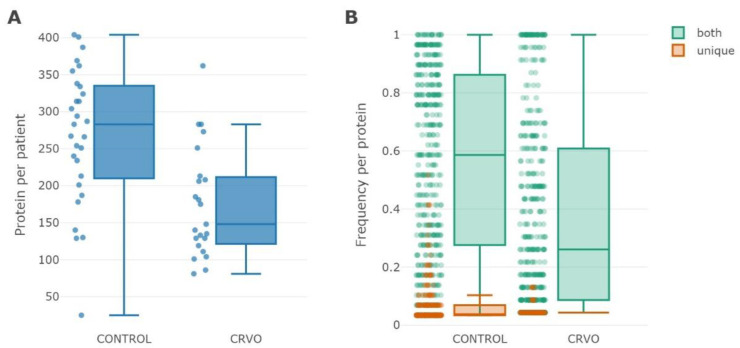
Box-plot of the proteome size (**A**) and frequency (**B**) distribution among subjects in the control and CRVO groups. The median size of individual proteins in the control group was found to be 283 (with a *q*1–*q*3 interquartile range between 210–335 proteins; maximal size was 404 proteins), whereas the median proteome size in the CRVO group was 148 proteins, with a *q*1–*q*3 interquartile range of 122–212 proteins and maximal size of 362 proteins (upper fence was 283 proteins). Blue dots indicate personal proteome size (**A**). Frequency distribution of proteins identified within the control and CRVO groups. The median frequency of the majority of group-specific proteins (brown color) was 0.03 (maximum frequency was 0.52, upper fence was 0.1) in the control group and 0.04 (maximum frequency was 0.13) among subjects with CRVO. Proteins of the shared part (green color) of the combined proteome featured a median frequency of 0.58 (*q*1–*q*3 interquartile range of 0.28–0.86) in the control group and a median frequency of 0.26 (*q*1–*q*3 interquartile range of 0.09–0.61) in the CRVO group (**B**).

In total, we accumulated 245,856 data-independent (DIA) mass spectra, of which 162,183 were classified as peptide spectra matches (PSMs) with an average accuracy of −1.2934 ± 3.3546 ppm. The collected PSMs covered 13,040 unique individual peptides. The proteomic analysis discovered 838 different proteins with at least two distinct peptides, of which 698 and 558 proteins were identified in the control and examined CRVO groups, respectively. The shared part of the proteome was comprised of 458 protein identifications with a frequency of more than 0.6. Despite the proportional size of the gathered proteomes within groups, the distribution of individual proteomes among subjects was more variable and made 283 proteins (median) within the interquartile range of *q*1 = 210 and *q*3 = 335 in the control group and 148 proteins (median) within the interquartile range of *q*1 = 122, *q*3 = 212) in the CRVO group (Figure 1A). Group-specific proteins were found to be the least frequent and consisted of 0.03 (median frequency value) and 0.04 amongst subjects in the control and CRVO groups, correspondingly (Figure 1B), while the median frequency of protein in the gathered proteome consisted of 0.58 and 0.26 in the control and CRVO groups, respectively.

**Figure 2:**
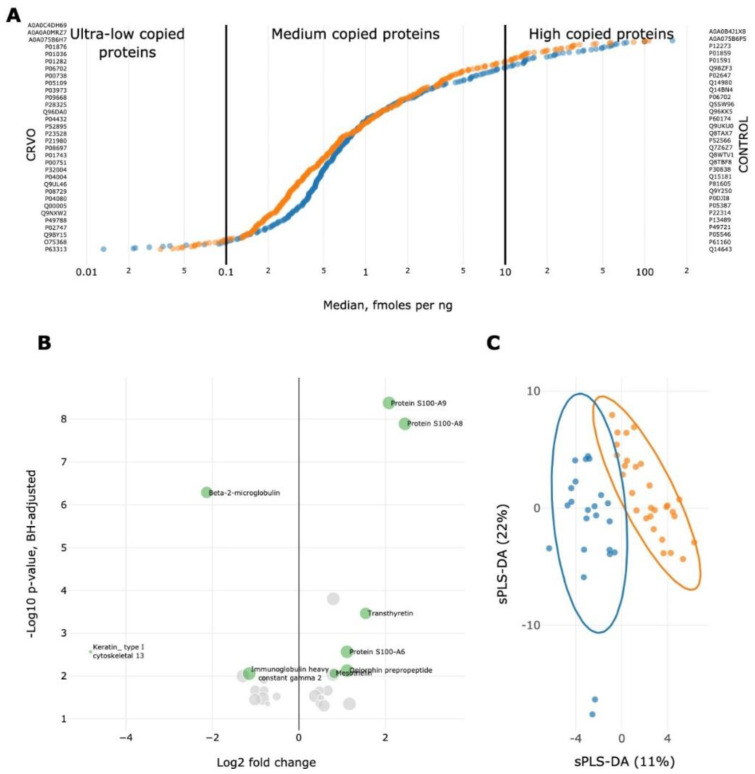
Distribution of protein quantitative properties in the combined proteome of control (orange color) and CRVO groups (blue color). The largest proportion (up to 78%) is covered by medium-copied proteins ranged between 0.1−10 fmoles/ng. The rest fraction can be split between low-copied (0.01−0.1 fmoles/ng) proteins (up to 8%) and high-copied (more than 10 fmoles/ng) proteins accounted up to 14% of the gathered tear proteome size. The listed proteins are ordered in a quantitative-dependent manner and arranged independently for the control (red color) and CRVO (blue color) groups (**A**). Volcano-plot scattering for 29 proteins (21 upregulated proteins (log_2_(FC) > 1) and 8 downregulated proteins (log_2_(FC) < −1) with a frequency of more than 0.6 (92% of proteins belonged to a frequency of 0.79−1.00) and significantly differed (*p* < 0.05, Wilcoxon test with Benjamini–Hochberg correction) between the control group and subjects with CRVO. Proteins that fit the FC and *p*-value criteria are highlighted by green (**B**). The sPLS-DA scatter plot displayed co-variances of 11% and 22% between CRVO subjects (blue color) and healthy donors (orange color) using common fraction of proteome (**C**).

In this study, we selected a combination of the UPS-2-based approach and matched peptides sum intensity as the most appropriate tool for the label-free quantification within the narrow range of concentrations [23]. A recent study demonstrated that employment of UPS-2 solely is not the most appropriate choice for quantitative proteomic measurements due to wide co-variation across the measured set of proteins. However, if UPS-2 is powered by one of the semi-quantitative techniques, including summed normalized intensity, the combined method achieves tighter standard deviations and better coefficient of variance (CV) values [24]. The UPS-2 set was used as an external standard instead of spiking into the sample because the majority of constituent proteins are of human origin. The CV value for UPS-2 in five replicates was quite satisfied (CV = 14.8% across 27 detected proteins) and fell with the expected value of accuracy [24]. Quantitative analysis demonstrated tight concentration-dependent distribution of measured proteins in both the CRVO and control groups (Figure 2A). It was found that proteins were distributed within four orders of magnitude and covered the range of concentrations between 0.01 and 500 fmoles/ng. The majority of proteins were medium-copied (between 0.1 and 10 fmoles/ng), whereas a small portion was split between low-copied (0.01–0.1 fmoles/ng) and high-copied (more than 10 fmoles/ng) in both studied groups (Figure 2A). The least abundant protein was found to be POTE ankyrin domain family member E (UniProt ID: Q6S8J3; 0.0037 fmoles/ng) in the control group, and in the CRVO group (0.1187 fmoles/ng); the most abundant proteins were Lactotransferrin (UniProt ID: P02788; 70.54 fmoles/ng) and Lysozyme C (UniProt ID: P61626; 108 fmoles/ng) in the CRVO and control groups, correspondingly (Supplementary Material). The obtained result of concentration-dependent protein distribution (Figure 2A) and similarity in the dimensionality of proteomes between groups suggests evenly distributed and proportional quantitative loading of proteins between studied groups. The listed proteins observed in both the CRVO and control groups and available for quantitative estimation (Figure 2A) were ordered in a quantitative-dependent manner and arranged independently for the control and CRVO groups, indicating a larger proportion of differing proteins in the medium-copied fraction and less proportion in the ultra-low- and high-copied proteins. The complete set of listed proteins and their measured concentrations is available in the Supplementary Material.

Performances of co-variable selection and classification to discriminate groups under consideration was achieved using sparse PLS-DA analysis, which displayed a variance of 11% and 22% between CRVO subjects and healthy donors (Figure 2C). To unveil the difference between study groups, we selected 137 mutual proteins with a frequency exceeding 0.6 in both the control and CRVO groups. Of them, only 29 proteins passed the significance cut-off (*p* < 0.05; Wilcoxon test with Benjamini–Hochberg correction), and this fraction was comprised of 8 downregulated (including three Ig-heavy and two Ig-light chains) and 21 upregulated proteins in the CRVO patients compared to healthy subjects (Table 1 and Figure 2B).

**Figure 3:**
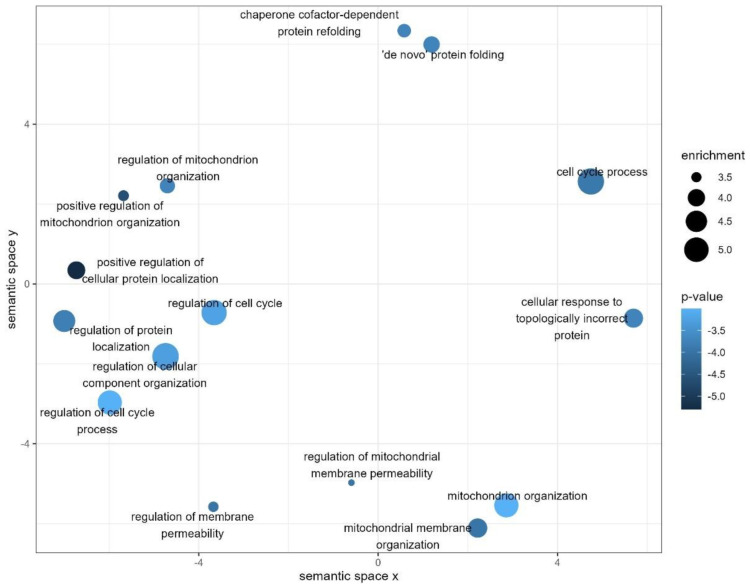
Distribution and the prevalence of GO biological process terms (filtered at *p* < 0.001) associated with the set of significantly different proteins. The semantic analysis is based on Resnik’s algorithm and measures the similarity between the enriched ontology terms after the elimination of redundant terms. The defined enrichment measure (circle size) defines relative abundance and the direction of a certain biological process within the study group (CRVO) relative to the control groups. The majority of processes attributed to cell cycle or mitochondria organization are significantly depleted or abolished, while the regulation of improperly folded proteins and stress response reaction are organized in the enhanced acting cluster.

The selected set of significantly differed proteins is characterized by a high frequency of distribution in the groups under consideration (92.1% of proteins have a frequency between 0.79 and 1.00 and the remaining proteins have a frequency between 0.6 and 0.78; Supplementary Material). The analysis of relevant biological processes (significance cut-off *p* < 0.001; Figure 3) showed that the majority of proteins are not highly specialized and implemented in the regulation of cellular response to misfolded proteins (*p* = 2.31 × 10^−4^), cellular response to topologically incorrect protein (*p* = 4.77 × 10^−4^), and positive regulation of mitochondrion organization (*p* = 9.91 × 10^−4^) processes. Molecular function mapping affirmed the prevalence of cell-stress-responsive proteins, and the main proportion of differed proteins are characterized by ubiquitin (*p* = 6.96 × 10^−6^), ubiquitin-like ligase binding activity (*p* = 1.49 × 10^−4^), and inhibition of peptidase and protease activity (*p* = 7.31 × 10^−4^).

The enrichment analysis revealed the prevalence of proteins involved in retinal homeostasis (GO:0001895; *p* = 6.06 × 10^−18^; FDR = 1.06 × 10^−13^), humoral immune response (GO:0006959; *p* = 6.18 × 10^−8^; FDR = 7.73 × 10^−5^), and defense response (GO:0006952; *p* = 3.39 × 10^−7^; FDR = 3.30 × 10^−4^). Obviously, the majority of such proteins are qualified as secreted (Supplementary Material) and can be found in tears and tear glands (*p* = 7.72 × 10^−15^), saliva (*p* = 4.65 × 10^−21^), bone marrow cells (*p* = 5.39 × 10^−11^), blood platelets (*p* = 2.44 × 10^−7^), submandibular glands (*p* = 9.25 × 10^−5^), and can be attributed as residents in right and left atrium tissue (*p* = 5.79 × 10^−5^). Due to the low tissue specificity and explicit multifunctionality, it is suggested that a large proportion of significantly differed proteins are poorly specialized, albeit they can play a pivotal role in numerous biological processes, particularly in immune response and retina hemostasis maintenance.

**Figure 4:**
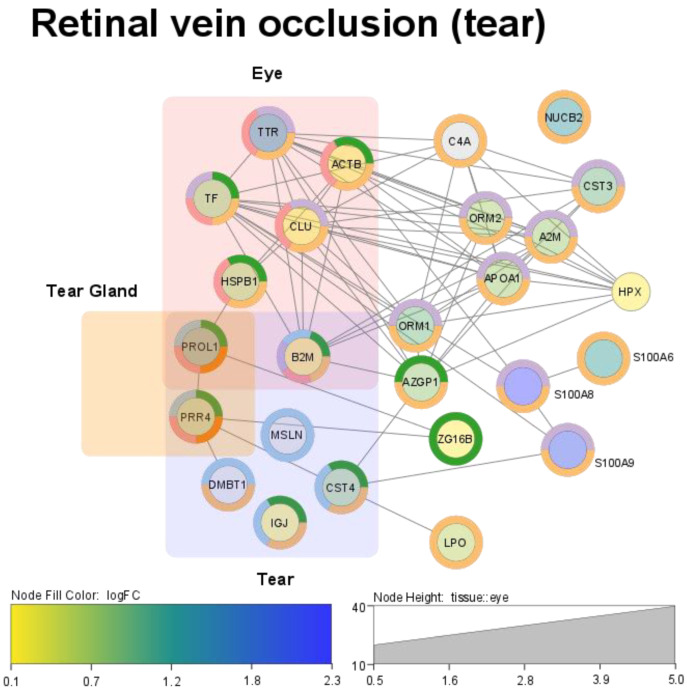
A map of critical interactions between the significantly different proteins. The map is reconstructed in the context of retinal vein occlusion (RVO) pathogenesis, tissue (source) specificity (such as tear gland, tears, and eye), and attributed with a quantitative property of measured proteins (color-scaled bar). The node height level indicates the confidence of protein interaction and normalized to eye-tissue specificity. Circle colors indicate the most prevalent biological processes that contribute to CRVO progression and are taken by mapped proteins (vesicle-mediated transport (magenta); defense response (cyan); retina hemostasis (green), regulation of immune system (orange), cell migration, and extracellular matrix organization (blue)). This figure has been produced in the Cytoscape software (version 3.8.2) for network data analysis, integration and visualization, and is available on the following link: https://cytoscape.org/ (accessed on 23 November 2022).

Protein–protein interaction analysis was conducted in support with Cytoscape using the STRING disease-related model, which is tightly associated with the retinal vein occlusion as a target pathology. The resulting network was percolated to eliminate missed interaction and those proteins falling below low molecular specialization. Ultimately, only proteins with a high confidence score (at least 0.7), implicated in retinal vein occlusion and attituded to eye tissue, tear gland, or tears, were selected to propose the final network of critical interactions (Figure 4) framed by the measured absolute quantitative properties (Table 1).

Despite the fact that the resulting proportion of network was small, almost the complete set of examined proteins had a high confidence (more than 0.7 confidence score) and was mapped in the CRVO-relevant network (Table 1). The designed network can be divided into four large clusters, of which three are with tissue-specific attributes (eye, tears, and tear gland), where proteins are produced by meibomian glands, lacrimal glands, and conjunctival goblet cells, and might indicate age- or disease-related changes in the tear proteome. The last cluster is the general cluster with multifunctional proteins but implicated in occlusion progression (Figure 4). The latter includes proteins of the extracellular matrix, blood, peroxisomes, and proteins involved in metabolic modulation (Table 1 and Supplementary Material).

**Figure 5:**
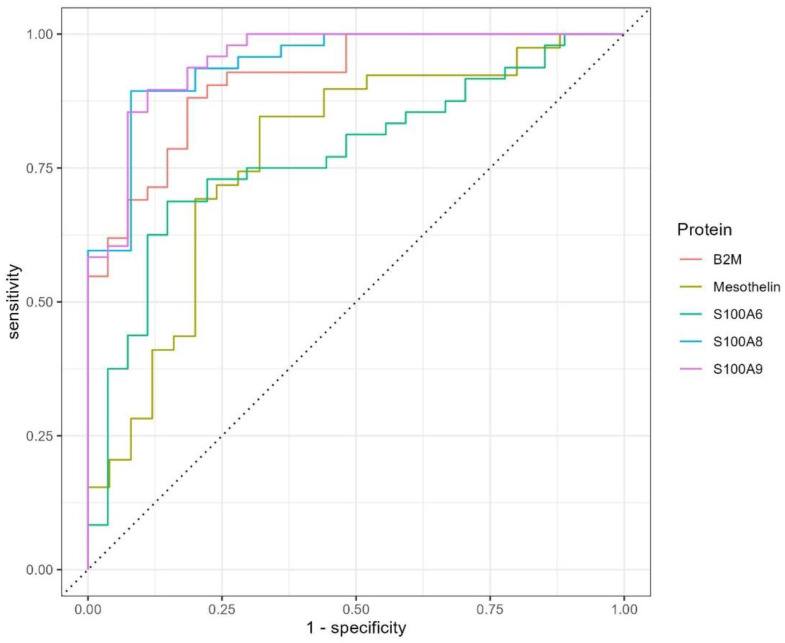
Receiver operating characteristic (ROC) plot of the area under the curve (AUC) for the most altered proteins found in the studied cohort and credibly associated with the CRVO pathology and the caused ischemic condition. The ROC curves have been plotted for S100A9 (AUC = 0.952), S100A8 (AUC = 0.945), B2M (AUC = 0.915), MSLN (AUC = 0.775), and S100A6 (AUC = 0.772).

To determine the significance of proteins implicated in oxidative stress and immune response, we estimated AUC measures using ROC plotting. We selected five proteins (S100A6, S100A8, S100A9, MSLN, and B2M) that met the criteria of the closest association with the CRVO and ischemic conditions, and were featured in the most significant alterations between the control group and subjects with CRVO (Figure 5).

The obtained results demonstrated satisfied AUC for each of the selected proteins. The AUC measure varied from the least estimated for S100A6 (AUC = 0.772) to a maximum value for S100A9 (AUC = 0.952). It should be noted that the largely contributing proteins (S100A8, S100A9, and B2M) belong to secreted proteins of the immune response and matrix-mediated regulation of cell migration, apoptosis regulation, and MHC-I representation. Despite the great specificity and sensitivity of the ROC curve (Figure 5), we avoided combining these proteins into the biomarker patterns, since much more examination is required to establish the proper role of the pattern in CRVO pathogenesis.

## 3. Discussion

Almost no overlapping proteins were found between our study and the top proteins established in tears of healthy subjects previously [25], apart from a few insignificant proteins (Lactotransferrin, Lipocalin-1, Lysozyme C, Complement C3; see Supplementary Materials) and one member of a significantly altered subgroup of proteins (Zinc-alpha-2-glycoprotein; Table 1). This observation may indicate the existing difference of the proteome content between healthy and affected or vulnerable subjects. However, these authors also reported a poor overlap (11.2%) with other studies and explained it as individual variability and challenges of the sample collection [19,25].

Besides age-related changes in the tear proteome, retinal vein occlusion (RVO) is accompanied by specific alterations in the matrix- and homeostasis-associated proteins. The impaired blood outflow is the pivotal cause of such changes, so the retina begins to suffer ischemic conditions upstream of the occlusion [26]. The ongoing anatomical changes induce an increasing level of VEGF and favor the inflammatory microenvironment [27].

A recent proteomic analysis of vitreous humor samples from RVO patients demonstrated upregulation of pro-inflammatory and immune-related proteins: C3 complement factor, CLU, VTN, and IGLL5 [28]. Although this small cohort of proteins is of interest, they merely indicate consequent undergoing processes and cannot be used as markers of retinal anatomical changes caused by occlusion. Analysis of an animal model with laser-induced BRVO established several stressing proteins that have not been reported before as being associated with RVO, and most of them were matrix-related (including laminin subunits, integrin, and actinin isoforms). Based on this evidence, the authors concluded that RVO is closely related to the matrix remodeling process, and focused on integrin and focal adhesion signaling [29]. Matrix-associated nectin-4 (PPR4), also known as proline-rich protein 4 (Table 1), responsible for focal cell adhesion, was also found among the upregulated subgroup of proteins in patients with CRVO, and was previously reported in diabetic retinopathy models [30].

Pro-inflammatory proteins of the tear proteome greatly contribute to distinguishing between various eye-related diseases [11]. Patients with proliferative and non-proliferative diabetic retinopathy are characterized by the abundance of S100A family proteins (S100A4, A100A6, S100A8, S100A11), but the most promising protein is S100A13 [31], which is the only S100A protein that has not been observed in our study as mentioned above (Supplementary Materials and Table 1). At the same time, a study of thyroid-associated orbitopathy revealed cystatin-C (CST4) among the top upregulated proteins [32], as we observed it for patients with CRVO (Table 1) among numerous defending proteins.

The S100 family proteins were rigorously reviewed in the context of eye diseases as a promising marker pattern with a high predictive potency and disease monitoring value [33,34]. These proteins have been repeatedly screened in tears and showed an ability to discriminate between age-related dry eye disease and CRVO due to controversial regulation (upregulated in CRVO) [11,35,36]. Our data suggests that ischemic conditions in CRVO patients might be associated with inflammation and mediated by immune-related proteins, since S100A family proteins and B2M (β2-microglobulin) (Table 1) are significantly upregulated.

These proteins construct a separate cluster on the customized disease-related map of protein–protein interactions (S100A6, S100A8, and S100A9; Figure 4) and are tightly joined with matrix-related (ACTB) and metal-binding (TF) proteins (Figure 4). Previously, a comparative study of CRVO and non-ischemic ocular disease patients revealed that the level of pro-inflammatory S100A family proteins directly correlates with the severity of retinal ischemia [37]. If S100A6 is a prolactin-receptor-associated protein mediating secretion of glucocorticoids, both S100A9 and S100A8 are regulators of cell migration and act in mechanisms against inflammation, oxidative stress, and stimulation of the innate immune response.

A proteomic study of patients with primary open angle glaucoma demonstrated a subgroup of 16 upregulated pro-inflammatory proteins [38], most of which were also observed in our study of CRVO patients as significantly altered, and include lactotransferrin, proline-rich-protein 4, zinc-α2-glycoprotein, and some immunoglobulin chains (Table 1). Moreover, the increased level of pro-inflammatory proteins is expectedly associated with the increased level of the defending proteins, of which lactoperoxidase and opiorphin prepropeptide displayed the most pronounced upregulation (Table 1) in patients with CRVO and patients with dry eye disease [39]. Hence, since typically pro-inflammatory proteins are observed together with an increased level of matrix-associated and defending proteins in tears of patients with eye diseases, one may conclude that these proteins indicate local inflammation as a consequence of occlusion and CRVO ischemic microenvironment.

Interestingly, a recent large-scale proteomic study of aqueous humor of patients with branch retinal vein occlusion (BRVO) found S100A8 and S100A9 were downregulated (FC = 0.30 at *p* = 0.030 and FC = 0.31 at *p* = 0.015, correspondingly), in opposition to CRVO (Table 1 and Supplementary Materials), but these alterations did not correlate directly with the central retinal thickness as a measure of severity [34]. The differences compared to the present data are, probably, caused by the source of the sample. The aqueous humor is an isolated and quite stable system and thereby changes caused by the ischemic condition are expectedly more responsive. Meanwhile, the same authors reported an about five-fold increase of pro-inflammatory S100A12 in the model of CRVO [11]. However, both investigations appreciate the importance of S100A proteins in the escalating inflammation under ischemic condition.

The cohort of secreted S100A proteins is well correlated with a significantly suppressed MHC-I peptide representing B2M protein (Table 1) and extracellular matrix proteins abundantly observed in our study, including PRR4, ACTB, CST4, CST3, and MSLN (Table 1), which presumably indicates the activation of chemokine pathways. It should be noted that MSLN (mesothelin) has not been ever reported regarding the tear proteome of either eye-related disease. The protein is generally mentioned as a target in the chemotherapy of different oncophenotypes, and mostly concerning solid tumors [40].

The finding of MSLN in the tear might be stressed, however, if one considers the increased level of extracellular matrix compounds as a pattern for CRVO and glaucoma [41,42,43]; the presence of MSLN seems reasonable due to its importance in cell adhesion as well as PPR4 and ACTB (Table 1). Presumably, the CRVO-related ischemic condition induces the expression of ocular-specific pro-fibrotic TGF-β2 factor, which sufficiently promotes the growth of extracellular matrix [43,44]. Thence, MSLN was consistently established among the upregulated subgroup of proteins in this study (Table 1). However, it should be admitted that matrix proteins are highly sensitive to oxidative stress and inflammation, whereas the primary sign is depicted in the maintenance of hemostasis and dysregulation of mitochondria functioning. It has been reported that patients with glaucoma and CRVO are characterized by irregular levels of GPX, SOD, and MDA, and typically present a higher level of these proteins even after surgery [45,46]. In addition, such proteins are frequently accompanied by a higher level of peptidase- and protease-inhibiting proteins, including CST4, OPRN, and LPO [38,39,47] as a consequence of retina homeostasis disruption and escalating immune reactivity, which agrees with the data obtained in this study (Table 1).

During macular degeneration, the association of oxidative stress with damaging and dysfunction of mitochondria is commonly observed [48]. Moreover, with aging, mitochondria become progressively more incomplete; thus, CRVO can be considered as an age-related disease with a pivotal role in the increase of ROS output [49]. In this study, oxidative stress is considered as a consequence of the suppression of mitochondria inner membrane organization processes (Figure 3), thus indicating the prevalence of apoptosis and cell damage. In contrast, processes related to the cell response to topologically incorrect proteins, chaperone–cofactor protein refolding and *de novo* protein folding (Figure 3 and Table 1), and inhibition of peptidase activity (OPRN, CST4; Table 1), are substantially upregulated in tears of patients with CRVO.

While all retinal cells rely on ATP as a fuel source and the photoreceptors are the largest consumers requiring regular mitochondria functioning, it can be hypothesized that such cautioning processes (Figure 3) underline the impaired retinal hemostasis and accompanied tissue damage caused by the excessive ROS generation. Furthermore, oxidative phosphorylation, which is stimulated by mitochondria, contributes significantly to the increased ATP and, consequently, ROS generation, and promotes the production of inflammatory cytokines [50]. Notwithstanding, only a few reports have established the relationship between oxidative stress and CRVO [45], but many more reports have reviewed the clinical relevance with manifested diabetic retinopathy [51,52,53].

If considering oxidative stress as the paramount background event of CRVO, it is essential to discover the role of ROS scavengers and the related transport system as a possible victim of the boosted ROS generation and cytokine production. The local increase of transthyretin (TTR), also known as prealbumin, was found in the proteome of CRVO patients with a high (0.88) prevalence (Table 1). The protein is mainly synthesized in the liver and also found in retinal pigment epithelial cells [54]. Besides the pivotal role in the transport of thyroxine to the brain, circulating TTR also carries retinol-binding proteins (RBPs) transporting and stabilizing retinol as an essential antioxidant agent. Due to its high toxic potency, retinol is primarily stored as fatty acid esters. Vitamin A can efficiently neutralize thiol-radicals and stabilize peroxide radicals, being an important factor that prevents and reduces the risk of oxidative stress and inflammation [55,56]. The deficiency of TTR may aggravate the risk of oxidative stress and enhance local impairment of blood flow and angiogenic processes, which is particularly established for cardiovascular and nondegenerative diseases [57]. On the other hand, the excess of circulating TTR provides a favorable environment for the transthyretin amyloidosis involved in numerous ophthalmological pathologies [54].

Despite most studies intending to assemble proteins in a panel of putative biomarkers, we are reluctant to shape the decision-making pattern of CRVO biomarkers. Instead, we focused on the five most significantly altered proteins, which are credibly associated with the pathogenesis of CRVO and capable of indicating the local ischemic condition and disruption of retinal homeostasis. We submitted S100A6, S100A8, S100A9, MSLN, and B2M proteins to estimate the area under the curve (Figure 5) and the resulting measures satisfy expectations of specificity and sensitivity for each protein under consideration, confirming their relationship to CRVO. Although some of these proteins are repeatedly mentioned in the context of CRVO severity and onset, their proper combination merits attention to find the very beginning of CRVO and to monitor the bases of ocular disease treatment.

## 4. Materials and Methods

### 4.1. General Workflow of the Study

This is a comparative study on relatively small cohorts of patients with CRVO and a relevant control group of healthy volunteers. Tear film liquid samples were collected and prepared for the discovery proteomic analysis in SONAR (data-independent mode) on a high-resolution quadrupole time-of-flight mass spectrometer. Confidently identified proteins were quantitatively assessed using Universal Proteomic Standard sample (UPS-2, Dynamic range) designed for quantitative proteomic assay [58]. The accumulated set of proteins were analyzed using a GO terms enrichment tool REVIGO [59] and Resnik’s semantic similarity approach [60]. Obtained results were enriched with quantitative measures and disease-related (CRVO) network interaction in Cytoscape (version 3.8.2). Finally, CRVO-related critical nodes and indications with quantitative levels were mapped.

### 4.2. Subjects and Ethical Consideration

Tear film liquid samples were obtained from *n* = 57 subjects, of which *n* = 28 were patients with central retinal vein occlusion (CRVO group) and *n* = 29 were healthy subjects of the control group (Table 2). Among patients with CRVO, *n* = 6 were diagnosed with incomplete occlusion and *n* = 22 with occlusion (Table 2). All subjects signed their informed consent to participate in the study. The study was approved by the Local Ethical Committee of the Russian Medical Academy of Continuous Professional Education (approval protocol no. 13 issued on 26 November 2019). The Approval was obtained in strict accordance with the tenets of the WMA Declaration of Helsinki on Biomedical Research Involving Human Subjects. All subjects were anonymized and no personal data can be disclosed with this paper. The detail of clinical records is presented in Table 2.

### 4.3. Sample Collection and Preparation

To minimize the ocular surface irritation, tear samples were obtained from the inferior temporal tear meniscus without anesthesia and collected from the affected eye using the calibrated volumetric glass microcapillary (10-µL nominal calibrated volume; Blaubrand, Wertheim, Germany) under the bright beam of light of a slit lamp. Samples were stored at −80 °C and transported in dry ice for proteomic analysis. In total, up to 100 µL of each tear sample was collected. Total protein fraction was measured using Pierce™ BCA Protein Assay Kit (Rockford, IL, USA) and made 5.8–7.7 mg/mL. Before alkylation and digestion of proteins, samples were aligned to 2.5 mg/mL in 35 mM triethylammonium bicarbonate (Sigma, Basel, Switzerland).

The tear sample, in a volume of 30 µL, was mixed with 120 µL of methanol (J. T. Baker; the Netherlands) for protein precipitation and centrifuged at 17,500× *g* acceleration at 15 °C for 10 min (Centrifuge 5424R, Eppendorf, Hamburg, Germany). The supernatant was discarded and the obtained precipitate was dissolved in 15 µL of the denaturation solution, consisting of 1% deoxycholic acid sodium salt (Sigma, Milan, Italy), 5 M urea (Sigma, Milan, Italy), 6% acetonitrile (Carlo Erba, Val-de-Reuil, France), and 300 mM sodium chloride (Fluka-Honeywell, Seelze, Germany), buffered by 75 mM triethylammonium bicarbonate to pH~8.5 (Sigma, Basel, Switzerland) and supplied with freshly prepared 10 mM tris-(2-carboxyethyl) phosphine (Sigma, St. Louis, MO, USA) for protein reduction. Samples were incubated for 20 min at 45 °C under continual stirring. Alkylation was supplied by the 2% solution of 4-vinylpyridine (Aldrich, Arklow, Ireland) in 30% isopropanol (Fisher Chemical, Loughborough, UK) added to 0.2% of the final concentration and incubated for 20 min at ambient temperature in darkness. Following alkylation, the sample was diluted ten times with triethylammonium bicarbonate 50 mM (pH 8.2). In-solution digestion with trypsin (Promega, Madison, WI, USA) resuspended to 200 ng/µL in 30 mM acetic acid (Carlo Erba, Val-de-Reuil, France) was performed in two consequent steps at a 1:50 (*w*/*w*) ratio for 3 h at 37 °C and at a 1:200 (*w*/*w*) ratio for 3 h at 42 °C. When digestion was completed, the reaction was quenched with 50% formic acid (Fisher Chemical, Loughborough, UK) added to a 2% final concentration. Samples were centrifuged at 12,500× *g* for 10 min at 10 °C to sediment insoluble deoxycholic acid. Ethylacetate (Carlo Erba, Val-de-Reuil, France) in a volume of 100 µL was added to clean the supernatant from deoxycholic acid remnants, shaken vigorously, and centrifuged at 12,500× *g* for 10 min at 10 °C again. The obtained clean supernatant was dried under a vacuum at 30 °C for 30–40 min, and the resulting pellet was reconstituted in 15 µL of 0.5% formic acid to obtain 1 µg/µL as the final estimated concentration of peptides considering initial aligned concentration of total protein fraction before sample preparation.

A Proteomics Dynamic Range Standard Set, or UPS-2 (Merck; St. Louis, MO, USA), that contains 48 proteins in a known concentration range covering five orders of magnitude, was used for the quantitative purpose of estimating protein concentration in the study cohort samples. Because the collected proteins (including recombinant) in the UPS-2 were preferably originated from human genome, there is a risk of inaccurate and incorrect estimation of protein concentration in tear samples. Therefore, the calibrating sample (UPS-2) was composed separately in a non-human matrix (*E. coli* strain K12) as described previously [61] and treated in the same manner as described above for tear samples. The calibrating sample was finally prepared in 10 µL to align the final concentration to be comparable with tear samples.

### 4.4. Liquid Chromatography and High-Resolution Mass Spectrometry Analysis

Samples were separated using an Acquity H-Class UPLC system (Waters, Elstree, UK) and loaded in a volume of 3 µL (totally 3 µg of peptides fraction on-column loading) onto an Acquity™ UPLC BEH C18 (2.1 × 50 mm, 1.7 µm particle size; Waters, Elstree, UK) column heated to 50 °C with the pre-installed in-line 0.2 µm filter, at a flow rate of 0.3 mL/min. Peptides were separated in a gradient of mobile phase A (water) and mobile phase B (acetonitrile), both supplied with 0.1% formic acid and 0.015% trifluoracetic acid using the following gradient scheme: 0–2.5 min 3% of B, then raising the B to 17% at 31.5 min, then raising the B to 37% at 45 min and rapid increasing of B to 97% at 47.5 min. The washing B was kept in the isocratic mode until 51 min at 0.45 mL/min flow rate, following smooth decreasing to the initial condition (3% of B) at 53.5 min and equilibration for the next 6 min at 0.3 mL/min.

Discovery proteomic analysis was performed on a high-resolution time-of-flight mass spectrometer Xevo G2-XS (Waters, Elstree, UK) equipped with a Z-spray electrostatic ionization source and operated in a positive ionization mode at a 2.8 kV capillary voltage and 85 V cone voltage with offset to 115 V. The desolvation gas flow rate was adjusted to 720 L/h at a temperature of 410 °C, and the cone gas flow was 50 L/h at a temperature of 150 °C. Precursor ions survey was conducted in a sensitivity analyzer mode within the 300–1250 *m/z* range within 235 ms of full duty cycle. Fragment ions were obtained after decomposition in CID mode at collision energy ramping within 14–42 eV, using argon as a collision. The acquisition was supported with an active online calibration by the lock-mass of *m/z* = 556.27 (Leu-Enkephalin at 50 pg/µL continually injected at a flow rate of 5 µL/min) applied every 30 s with isolation tolerance of 3 mDa.

### 4.5. Proteomic Data Analysis

Raw data files were processed using PLGS (Protein Lynx Global Server, version 3.0.3, Waters, Elstree, UK) search engine against a human protein amino acid sequences database obtained from UniProt KB (release May 2021) as a FASTA file enriched by the sequence of recombinant BH3-interacting domain death agonist from the UPS-2 (the FASTA of UPS-2 set proteins is available on http://www.sigma.com/ups accessed on 23 November 2022). Reversed concatenated decoy sequences were added to the database automatically to estimate a false positive rate. The search was performed at a precursor mass tolerance of 20 ppm (±10 ppm tolerance window) and a fragment mass tolerance of 0.008 Da (±4 mDa tolerance window). Searching included *S*-pyridilethylation as a fixed modification, and oxidized methionine and Q/N deamidation as variable modifications. The minimal peptide length was fixed to at least eight amino acid residues, and only one internal missed cleavage was allowed. The false discovery rate (FDR) at 1% was determined for peptide and protein identification by accumulating the reverse database hits. 

The quantitative measurement has been performed by applying the Creatine kinase M-type (P06732 UniProt accession number) and BH3-interacting domain death agonist (P55957 UniProt accession number) included in the UPS-2 set at 500 fmoles and 50 fmoles amounts (or 50 fmoles/µL and 5 fmoles/µL in the final calibrating UPS-2 sample of 10 µL volume), respectively, which corresponds to 150 fmoles and 15 fmoles of Creatine kinase M-type and BH3-interacting domain death agonist loaded on the column (three µL of loading volume), or 0.05 fmoles/ng and 0.005 fmoles/ng, correspondingly. The quantitative analysis was performed in the PLGS environment and based on the combination of the UPS-2-based calibration and matched peptides sum intensity as one of the reliable approaches for label-free quantitation [24] in samples with a comparatively narrow (up to five orders of magnitude) range of concentrations.

The mass spectrometry proteomics data have been deposited to the ProteomeXchange Consortium via the PRIDE [62] partner repository with the dataset identifier PXD037021 and 10.6019/PXD037021.

### 4.6. Statistical Data Analysis

Acceptance criteria for the protein identification were based on the Human Proteome Project Mass Spectrometry Data Interpretation Guidelines 3.0 [63], but were applied in a more flexible manner. At least two distinct-by-sequence peptides, of which one must be a unique (proteotypic) peptide (apart from isoform-specific peptides), were accepted for regular protein identification. A fraction of criteria-met proteins was extracted for the statistical analysis following the protein identification. To achieve data reduction and discrimination, and perform variable selection and classification of the studied cohort, sparse partial least-squares discriminant analysis (sPLS-DA) with 0.95 ellipse area confidence level was utilized. Protein intensity was estimated as the normalized summed peptide intensities belonging to a certain protein, and the resulting matrix of intensities was used for the quantification based on the calculation of intensity medians for each protein within studied groups. Wilcoxon test (*p* < 0.05) with Benjamini–Hochberg correction for multiple hypothesis testing was applied to the study groups to reveal outliers and significant differences in quantitative loading. Proteins with no concentration measure were imputated and not considered for estimation of between-groups difference. A measure of protein abundance was represented as a median value fold change (FC) ratio toward the control group and calculated based on the absolute concentration sampled from the UPS-2-based quantitative analysis. Proteins with a frequency of at least 0.6 within a group, and beyond the fold change cut-offs of >2 or <0.5 at a *p*-value less than 0.05, were considered as significant in quantitative property. Statistical values were calculated in the R (version 3.2.0) environment.

Significantly altered proteins were submitted for the functional and pathway analysis at a *q*-value threshold less than *q* < 0.01 using the Cytoscape (version 3.8.2) software. Pathway and network reconstruction were performed using a STRING (version 11.5) database query in the disease-related mode with a confidence score of 0.7 by employing the retinal vein occlusion-related proteins open access network as a source for the network customization and reconstruction. Both customized after protein identification and disease-related (CRVO) networks we gathered to consolidate the final reconstruction of CRVO-associated interactions. The consolidated network was enriched by the quantitative properties of the identified proteins and repopulated in the Cytoscape by applying the FDR < 0.01 percolation filter. Proteins enriched by the GO terms of biological processes (GO database release of September 2021) were rendered with a similarity coefficient of more than 0.7 to refine the protein interaction networks. The tissue filter option of the Cytoscape was applied to group relevant proteins into disease-related reconstructed networks by their tissue representation, which is estimated based on the transcriptomic, antibody and proteomic data collected by the STRING. Analysis of biological processes direction and elimination of redundant GO-terms attributed to the enriched protein was performed in support with REVIGO [59] (GO database update of 2 July 2021; UniProt-to-GO mapping database update of 17 July 2021) at a *p* < 0.001 and Resnik’s semantic similarity approach, which measures similarity between two terms as simply the information content of their most informative common ancestor [60]. Area under the curve (AUC) using a ROC operator was plotted for the most significantly altered proteins taken from the studied group and associated with the CRVO to determine the meaningfulness of protein specificity and sensitivity to certain pathology. All statistical calculations were performed using R Statistical Software (v4.1.2; R Core Team 2021).

## 5. Conclusions

We found a large proportion (458 proteins) of tear proteomes common for both patients with CRVO and the control group. Among proteins selected for quantitative analysis, a neglected portion overlapped with studies focused on the tears of healthy donors [25], but more a promising (up to 86%) fraction was observed with studies of retinopathy [31] and thyroid-associated orbitopathy [32]. The group of 29 proteins was determined as up- or downregulated and attributed to the immune response, inflammation, mitochondrial dysfunction and retina homeostasis. It is assumed that the profound decrease of B2M and immediate increase of S100A proteins with the abundance of matrix-associated proteins underlines cell migration and cytokine production caused by the progressing ischemic condition. The prevalence of mesothelin (MSLN) indicates the ongoing matrix remodeling due to its importance in the positive regulation of cell adhesion. Although the protein has never been reported in tear liquid proteome before, it is well recognized that MSLN is stimulated by the ocular-specific pro-fibrotic TGF-β2 factor.

Mitochondria maintenance proteins are the most sensitive indicators in response to escalating oxidative stress. It was determined that processes associated with mitochondria biogenesis and mitochondria inner membrane formation were significantly suppressed in CRVO patients, whereas processes associated with protein metabolism (*de novo* folding, chaperon cofactor-dependent refolding, cellular response to topologically incorrect proteins), in contrast, were the most prevalent.

The main limitation of this study is the insufficient size of population under the study. By tacit assumption, small population dimensionality may affect the final results and accuracy of conclusion. However, our findings match previously reported conclusions regarding the role of S100A proteins in the pathogenesis of CRVO [34] and their increase under ischemic conditions [11] in dry age-related macular degeneration and diabetic retinopathy [9,26].

In summary, we assume that there are several promising proteins capable of highlight new insights into our understating of CRVO pathology, but their utility in part or in whole is questionable until multiple examinations have been provided.

## Figures and Tables

**Table 1:** Quantitative assessment of the common part of the proteome (*p* < 0.05). Due to the frequency percolation (more than 0.6 value within each group) being applied before the selection of significant proteins, the estimated concentration is designed as a median value with an interquartile range (IQR). The protein concentration is estimated as an amount of certain protein (in fmoles) per one ng of total protein on-column loading (fmoles/ng). The total amount of protein on-column loading was 3 (three) µg per sample. Proteins falling off the FC cut-off level (log_2_(FC) > 1 or log_2_(FC) < −1) are color filled.

Identifiers			Frequency		Concentration, fmoles/ng		FC log2	*p*-Value
Accession	Gene	Name	CRVO	Control	CRVO	Control		
P61769	B2M	β2-microglobulin	0.91	0.9	2.29 ± 1.72	10.04 ± 10.34	−2.13	1.49 × 10^−10^
P60709	ACTB	Actin cytoplasmic 1	1	1	5.73 ± 8.81	14.06 ± 8.17	−1.3	4.67 × 10^−3^
P01859	IGHG2	Immunoglobulin heavy γ2	1	1	6.37 ± 11.42	14.12 ± 12.43	−1.15	7.62 × 10^−4^
Q96DA0	ZG16B	Zymogen granule protein 16 homolog B	0.83	0.72	1.29 ± 0.87	2.6 ± 3.26	−1.01	3.86 × 10^−3^
P01860	IGHG3	Immunoglobulin heavy γ3	1	0.97	0.56 ± 0.87	1 ± 1.63	−0.83	2.20 × 10^−2^
P02790	HPX	Hemopexin	0.91	0.97	2.48 ± 2.58	3.52 ± 4.71	−0.51	1.23 × 10^−2^
A0A0A0MRZ7	IGKV2D−26	Immunoglobulin κ-variable 2D-26	0.61	0.76	1.7 ± 0.68	2.32 ± 1.86	−0.45	2.14 × 10^−2^
P0DOY3	IGLC3	Immunoglobulin λ3	1	1	27.37 ± 17.28	34.2 ± 20.03	−0.32	1.12 × 10^−2^
P01591	JCHAIN	Immunoglobulin J chain	1	1	11.03 ± 8.44	10.97 ± 15.47	0.01	4.00 × 10^−2^
P04792	HSPB1	Heat shock protein β1	0.96	0.97	1.62 ± 2.87	1.53 ± 1.22	0.08	2.13 × 10^−2^
P10909	CLU	Clusterin	1	0.97	5.07 ± 4.28	4.37 ± 4.94	0.21	9.48 × 10^−3^
P02647	APOA1	Apolipoprotein A-I	1	0.97	5.57 ± 12.16	4.18 ± 7.12	0.42	3.93 × 10^−2^
Q16378	PRR4	Proline-rich protein 4	0.65	0.76	41.4 ± 40.03	30.01 ± 25.78	0.46	1.22 × 10^−2^
P22079	LPO	Lactoperoxidase	0.74	0.66	0.73 ± 0.66	0.52 ± 0.29	0.47	1.61 × 10^−2^
P0C0L4	C4A	Complement C4-A	0.61	0.69	0.81 ± 0.42	0.57 ± 0.58	0.52	1.93 × 10^−3^
P01034	CST3	Cystatin-C	0.91	0.62	1.22 ± 0.9	0.81 ± 1.1	0.58	8.46 × 10^−3^
P19652	ORM2	α1-acid glycoprotein 2	1	0.93	1.64 ± 1.69	1.09 ± 1.4	0.59	3.97 × 10^−2^
P01023	A2M	α2-macroglobulin	0.65	0.83	1.54 ± 1.12	0.99 ± 0.97	0.64	1.32 × 10^−2^
Q9UGM3	DMBT1	Deleted in malignant brain tumors 1 protein	0.83	0.97	11.41 ± 11.35	7.18 ± 6.87	0.67	1.82 × 10^−3^
P02787	TF	Serotransferrin	1	1	13.91 ± 14.94	8.73 ± 10.61	0.67	2.56 × 10^−2^
P01036	CST4	Cystatin-S	1	0.97	22.82 ± 10.7	13.24 ± 12.76	0.79	1.61 × 10^−3^
P25311	AZGP1	Zinc-α2-glycoprotein	1	1	49.07 ± 10.71	28.26 ± 18.61	0.8	2.02 × 10^−7^
Q13421	MSLN	Mesothelin	0.78	0.83	0.95 ± 0.59	0.54 ± 0.44	0.82	1.44 × 10^−4^
P06703	S100A6	Protein S100-A6	1	0.9	8.62 ± 11.01	3.99 ± 2.28	1.11	6.00 × 10^−5^
Q99935	OPRPN	Opiorphin prepropeptide	1	0.97	49.38 ± 25.83	22.82 ± 29.03	1.11	5.92 × 10^−4^
P02763	ORM1	α1-acid glycoprotein 1	1	0.97	7.68 ± 9.04	3.41 ± 5.3	1.17	3.19 × 10^−2^
P02766	TTR	Transthyretin	0.91	0.52	0.81 ± 0.78	0.28 ± 0.26	1.54	8.87 × 10^−6^
P06702	S100A9	Protein S100-A9	1	0.9	8.78 ± 17.08	2.07 ± 1.08	2.08	9.50 × 10^−14^
P05109	S100A8	Protein S100-A8	1	0.86	3.74 ± 7.48	0.68 ± 0.62	2.45	1.75 × 10^−12^

**Table 2:** Main clinical and anthropometric characteristics of subjects of CRVO group and relevant control group. Definitions in: OD—Ocular Dexter (right eye), OS—Oculus Sinister (left eye), OU—Oculus Uterque (both eyes).

Parameter	CRVO Group			Control Group	*p*-Value
Population size	28			29	-
Gender ratio (male/female)	15/13 (53%/47%)			17/12 (58%/42%)	0.80 **^‡^**
Diagnosis	OD	OS	OU	-	-
Incomplete occlusion	4	2	-		
Occlusion (Thrombosis)	10	10	2		
Age, average (±SD), years old	71.7 ± 5.6			75.1 ± 7.1	0.47 ^#^
Myocardial ischemia	10			6	-
Diabetes mellitus (type 2)	2			-	
Hypertensive disease	26			27	
Anticoagulant medication	11			-	
Central retinal thickness, µm ^†^	282 ± 167			157 ± 39	
Total cholesterol level, mmol/L					
less than 5 mmol/L	4 (14%)			29	-
5–7 mmol/L	17 (61%)			-	
more than 7 mmol/L	7 (25%)			-	

**^‡^** Chi-squared test; ^#^ Wilcoxon rank-sum test; ^†^ central retinal thickness (CRT) has been obtained only for 22 of 28 subjects in the CRVO group and for 20 of 29 subjects in the control group.

## Data Availability

Data are available via ProteomeXchange with identifier PXD037021 and https://doi.org/10.6019/PXD037021.

## Supplementary Materials

The following supporting information can be downloaded at: https://www.mdpi.com/article/10.3390/ijms232314653/s1.

## References

[1] Song P., Xu Y., Zha M., Zhang Y., Rudan I. (2019). Global epidemiology of retinal vein occlusion: A systematic review and meta-analysis of prevalence, incidence, and risk factors. J. Glob. Health.

[2] Laouri M., Chen E., Looman M., Gallagher M. (2011). The burden of disease of retinal vein occlusion: Review of the literature. Eye.

[3] Cugati S., Wang J.J., Rochtchina E., Mitchell P. (2006). Ten-year incidence of retinal vein occlusion in an older population: The Blue Mountains Eye Study. Arch. Ophthalmol..

[4] Mir T.A., Kherani S., Hafiz G., Scott A.W., Zimmer-Galler I., Wenick A.S., Solomon S., Han I., Poon D., He L. (2016). Changes in Retinal Nonperfusion Associated with Suppression of Vascular Endothelial Growth Factor in Retinal Vein Occlusion. Ophthalmology.

[5] Bhandari S., Nguyen V., Hunt A., Gabrielle P.-H., Viola F., Mehta H., Manning L., Squirrell D., Arnold J., McAllister I.L. (2022). Changes in 12-month outcomes over time for age-related macular degeneration, diabetic macular oedema and retinal vein occlusion. Eye.

[6] Kang E.Y.-C., Yeung L., Lee Y.-L., Wu C.-H., Peng S.-Y., Chen Y.-P., Gao Q.-Z., Lin C., Kuo C.-F., Lai C.-C. (2021). A Multimodal Imaging-Based Deep Learning Model for Detecting Treatment-Requiring Retinal Vascular Diseases: Model Development and Validation Study. JMIR Med. Inform..

[7] Yong H., Qi H., Yan H., Wu Q., Zuo L. (2021). The correlation between cytokine levels in the aqueous humor and the prognostic value of anti-vascular endothelial growth factor therapy for treating macular edema resulting from retinal vein occlusion. Graefe’s Arch. Clin. Exp. Ophthalmol..

[8] Jung S.H., Kim K.-A., Sohn S.W., Yang S.J. (2014). Association of aqueous humor cytokines with the development of retinal ischemia and recurrent macular edema in retinal vein occlusion. Invest. Ophthalmol. Vis. Sci..

[9] Yuan X., Gu X., Crabb J.S., Yue X., Shadrach K., Hollyfield J.G., Crabb J.W. (2010). Quantitative proteomics: Comparison of the macular Bruch membrane/choroid complex from age-related macular degeneration and normal eyes. Mol. Cell. Proteom..

[10] Kotwal R.S., Butler F.K.J., Murray C.K., Hill G.J., Rayfield J.C., Miles E.A. (2009). Central retinal vein occlusion in an Army Ranger with glucose-6-phosphate dehydrogenase deficiency. J. Spec. Oper. Med..

[11] Cehofski L.J., Kruse A., Kirkeby S., Alsing A.N., Ellegaard Nielsen J., Kojima K., Honoré B., Vorum H. (2018). IL-18 and S100A12 Are Upregulated in Experimental Central Retinal Vein Occlusion. Int. J. Mol. Sci..

[12] Vogl T., Pröpper C., Hartmann M., Strey A., Strupat K., van den Bos C., Sorg C., Roth J. (1999). S100A12 is expressed exclusively by granulocytes and acts independently from MRP8 and MRP14. J. Biol. Chem..

[13] Zhang Y., Cho C.-H., Atchaneeyasakul L., McFarland T., Appukuttan B., Stout J.T. (2005). Activation of the mitochondrial apoptotic pathway in a rat model of central retinal artery occlusion. Invest. Ophthalmol. Vis. Sci..

[14] Nordgaard C.L., Karunadharma P.P., Feng X., Olsen T.W., Ferrington D.A. (2008). Mitochondrial proteomics of the retinal pigment epithelium at progressive stages of age-related macular degeneration. Invest. Ophthalmol. Vis. Sci..

[15] Xu H.-J., Li Q.-Y., Zou T., Yin Z.-Q. (2021). Development-related mitochondrial properties of retinal pigment epithelium cells derived from hEROs. Int. J. Ophthalmol..

[16] Nordgaard C.L., Berg K.M., Kapphahn R.J., Reilly C., Feng X., Olsen T.W., Ferrington D.A. (2006). Proteomics of the retinal pigment epithelium reveals altered protein expression at progressive stages of age-related macular degeneration. Invest. Ophthalmol. Vis. Sci..

[17] Zecha J., Satpathy S., Kanashova T., Avanessian S.C., Kane M.H., Clauser K.R., Mertins P., Carr S.A., Kuster B. (2019). TMT Labeling for the Masses: A Robust and Cost-efficient, In-solution Labeling Approach. Mol. Cell. Proteom..

[18] Poulos R.C., Hains P.G., Shah R., Lucas N., Xavier D., Manda S.S., Anees A., Koh J.M.S., Mahboob S., Wittman M. (2020). Strategies to enable large-scale proteomics for reproducible research. Nat. Commun..

[19] Bachhuber F., Huss A., Senel M., Tumani H. (2021). Diagnostic biomarkers in tear fluid: From sampling to preanalytical processing. Sci. Rep..

[20] Noma H., Funatsu H., Mimura T., Tatsugawa M., Shimada K., Eguchi S. (2012). Vitreous inflammatory factors and serous macular detachment in branch retinal vein occlusion. Retina.

[21] Noma H., Funatsu H., Sakata K., Mimura T., Hori S. (2010). Association between macular microcirculation and soluble intercellular adhesion molecule-1 in patients with macular edema and retinal vein occlusion. Graefe’s Arch. Clin. Exp. Ophthalmol..

[22] Cehofski L.J., Kruse A., Bøgsted M., Magnusdottir S.O., Stensballe A., Honoré B., Vorum H. (2016). Retinal proteome changes following experimental branch retinal vein occlusion and intervention with ranibizumab. Exp. Eye Res..

[23] Muntel J., Kirkpatrick J., Bruderer R., Huang T., Vitek O., Ori A., Reiter L. (2019). Comparison of Protein Quantification in a Complex Background by DIA and TMT Workflows with Fixed Instrument Time. J. Proteome Res..

[24] Millán-Oropeza A., Blein-Nicolas M., Monnet V., Zivy M., Henry C. (2022). Comparison of Different Label-Free Techniques for the Semi-Absolute Quantification of Protein Abundance. Proteomes.

[25] Dor M., Eperon S., Lalive P.H., Guex-Crosier Y., Hamedani M., Salvisberg C., Turck N. (2019). Investigation of the global protein content from healthy human tears. Exp. Eye Res..

[26] Cehofski L.J., Honoré B., Vorum H. (2017). A Review: Proteomics in Retinal Artery Occlusion, Retinal Vein Occlusion, Diabetic Retinopathy and Acquired Macular Disorders. Int. J. Mol. Sci..

[27] Campochiaro P.A., Hafiz G., Mir T.A., Scott A.W., Sophie R., Shah S.M., Ying H.S., Lu L., Chen C., Campbell J.P. (2015). Pro-Permeability Factors After Dexamethasone Implant in Retinal Vein Occlusion; the Ozurdex for Retinal Vein Occlusion (ORVO) Study. Am. J. Ophthalmol..

[28] Reich M., Dacheva I., Nobl M., Siwy J., Schanstra J.P., Mullen W., Koch F.H.J., Kopitz J., Kretz F.T.A., Auffarth G.U. (2016). Proteomic Analysis of Vitreous Humor in Retinal Vein Occlusion. PLoS ONE.

[29] Cehofski L.J., Kruse A., Kjærgaard B., Stensballe A., Honoré B., Vorum H. (2015). Proteins involved in focal adhesion signaling pathways are differentially regulated in experimental branch retinal vein occlusion. Exp. Eye Res..

[30] Nishikawa T., Giardino I., Edelstein D., Brownlee M. (2000). Changes in diabetic retinal matrix protein mRNA levels in a common transgenic mouse strain. Curr. Eye Res..

[31] Amorim M., Martins B., Caramelo F., Gonçalves C., Trindade G., Simão J., Barreto P., Marques I., Leal E.C., Carvalho E. (2022). Putative Biomarkers in Tears for Diabetic Retinopathy Diagnosis. Front. Med..

[32] Kishazi E., Dor M., Eperon S., Oberic A., Hamedani M., Turck N. (2018). Thyroid-associated orbitopathy and tears: A proteomics study. J. Proteom..

[33] Tamhane M., Cabrera-Ghayouri S., Abelian G., Viswanath V. (2019). Review of Biomarkers in Ocular Matrices: Challenges and Opportunities. Pharm. Res..

[34] Cehofski L.J., Kojima K., Terao N., Kitazawa K., Thineshkumar S., Grauslund J., Vorum H., Honoré B. (2020). Aqueous Fibronectin Correlates With Severity of Macular Edema and Visual Acuity in Patients With Branch Retinal Vein Occlusion: A Proteome Study. Invest. Ophthalmol. Vis. Sci..

[35] Srinivasan S., Thangavelu M., Zhang L., Green K.B., Nichols K.K. (2012). iTRAQ quantitative proteomics in the analysis of tears in dry eye patients. Invest. Ophthalmol. Vis. Sci..

[36] Ma J.Y.W., Sze Y.H., Bian J.F., Lam T.C. (2021). Critical role of mass spectrometry proteomics in tear biomarker discovery for multifactorial ocular diseases (Review). Int. J. Mol. Med..

[37] Noma H., Funatsu H., Mimura T. (2012). Vascular endothelial growth factor and interleukin-6 are correlated with serous retinal detachment in central retinal vein occlusion. Curr. Eye Res..

[38] Pieragostino D., Agnifili L., Fasanella V., D’Aguanno S., Mastropasqua R., Di Ilio C., Sacchetta P., Urbani A., Del Boccio P. (2013). Shotgun proteomics reveals specific modulated protein patterns in tears of patients with primary open angle glaucoma naïve to therapy. Mol. Biosyst..

[39] Giannaccare G., Ghelardini C., Mancini A., Scorcia V., Di Cesare Mannelli L. (2021). New Perspectives in the Pathophysiology and Treatment of Pain in Patients with Dry Eye Disease. J. Clin. Med..

[40] Hassan R., Thomas A., Alewine C., Le D.T., Jaffee E.M., Pastan I. (2016). Mesothelin Immunotherapy for Cancer: Ready for Prime Time?. J. Clin. Oncol. Off. J. Am. Soc. Clin. Oncol..

[41] Knepper P.A., Mayanil C.S.K., Goossens W., Wertz R.D., Holgren C., Ritch R., Allingham R.R. (2002). Aqueous humor in primary open-angle glaucoma contains an increased level of CD44S. Invest. Ophthalmol. Vis. Sci..

[42] Agarwal P., Daher A.M., Agarwal R. (2015). Aqueous humor TGF-β2 levels in patients with open-angle glaucoma: A meta-analysis. Mol. Vis..

[43] Igarashi N., Honjo M., Yamagishi R., Kurano M., Yatomi Y., Igarashi K., Kaburaki T., Aihara M. (2021). Crosstalk between transforming growth factor β-2 and Autotaxin in trabecular meshwork and different subtypes of glaucoma. J. Biomed. Sci..

[44] Picht G., Welge-Luessen U., Grehn F., Lütjen-Drecoll E. (2001). Transforming growth factor beta 2 levels in the aqueous humor in different types of glaucoma and the relation to filtering bleb development. Graefe’s Arch. Clin. Exp. Ophthalmol..

[45] Masuda T., Shimazawa M., Hara H. (2017). Retinal Diseases Associated with Oxidative Stress and the Effects of a Free Radical Scavenger (Edaravone). Oxid. Med. Cell. Longev..

[46] Flammer J., Konieczka K., Bruno R.M., Virdis A., Flammer A.J., Taddei S. (2013). The eye and the heart. Eur. Heart J..

[47] Barka T., Asbell P.A., van der Noen H., Prasad A. (1991). Cystatins in human tear fluid. Curr. Eye Res..

[48] Zorov D.B., Juhaszova M., Sollott S.J. (2014). Mitochondrial reactive oxygen species (ROS) and ROS-induced ROS release. Physiol. Rev..

[49] Davalli P., Mitic T., Caporali A., Lauriola A., D’Arca D. (2016). ROS, Cell Senescence, and Novel Molecular Mechanisms in Aging and Age-Related Diseases. Oxid. Med. Cell. Longev..

[50] Juel H.B., Faber C., Svendsen S.G., Vallejo A.N., Nissen M.H. (2013). Inflammatory cytokines protect retinal pigment epithelial cells from oxidative stress-induced death. PLoS ONE.

[51] Kowluru R.A. (2017). Diabetic retinopathy, metabolic memory and epigenetic modifications. Vis. Res..

[52] Kowluru R.A. (2020). Diabetic Retinopathy: Mitochondria Caught in a Muddle of Homocysteine. J. Clin. Med..

[53] Kowluru R.A. (2013). Mitochondria damage in the pathogenesis of diabetic retinopathy and in the metabolic memory associated with its continued progression. Curr. Med. Chem..

[54] Minnella A.M., Rissotto R., Antoniazzi E., Di Girolamo M., Luigetti M., Maceroni M., Bacherini D., Falsini B., Rizzo S., Obici L. (2021). Ocular Involvement in Hereditary Amyloidosis. Genes.

[55] Palace V.P., Khaper N., Qin Q., Singal P.K. (1999). Antioxidant potentials of vitamin A and carotenoids and their relevance to heart disease. Free Radic. Biol. Med..

[56] Carazo A., Macáková K., Matoušová K., Krčmová L.K., Protti M., Mladěnka P. (2021). Vitamin A Update: Forms, Sources, Kinetics, Detection, Function, Deficiency, Therapeutic Use and Toxicity. Nutrients.

[57] Kaysen G.A., Eiserich J.P. (2004). The role of oxidative stress-altered lipoprotein structure and function and microinflammation on cardiovascular risk in patients with minor renal dysfunction. J. Am. Soc. Nephrol..

[58] Sánchez B.J., Lahtvee P.-J., Campbell K., Kasvandik S., Yu R., Domenzain I., Zelezniak A., Nielsen J. (2021). Benchmarking accuracy and precision of intensity-based absolute quantification of protein abundances in Saccharomyces cerevisiae. Proteomics.

[59] Supek F., Bošnjak M., Škunca N., Šmuc T. (2011). REVIGO summarizes and visualizes long lists of gene ontology terms. PLoS ONE.

[60] Pesquita C., Faria D., Falcão A.O., Lord P., Couto F.M. (2009). Semantic similarity in biomedical ontologies. PLoS Comput. Biol..

[61] Kopylov A.T., Kaysheva A.L., Papysheva O., Gribova I., Kotaysch G., Kharitonova L., Mayatskaya T., Krasheninnikova A., Morozov S.G. (2020). Association of Proteins Modulating Immune Response and Insulin Clearance During Gestation with Antenatal Complications in Patients with Gestational or Type 2 Diabetes Mellitus. Cells.

[62] Perez-Riverol Y., Bai J., Bandla C., García-Seisdedos D., Hewapathirana S., Kamatchinathan S., Kundu D.J., Prakash A., Frericks-Zipper A., Eisenacher M. (2022). The PRIDE database resources in 2022: A hub for mass spectrometry-based proteomics evidences. Nucleic Acids Res..

[63] Deutsch E.W., Lane L., Overall C.M., Bandeira N., Baker M.S., Pineau C., Moritz R.L., Corrales F., Orchard S., Van Eyk J.E. (2019). Human Proteome Project Mass Spectrometry Data Interpretation Guidelines 3.0. J. Proteome Res..

